# Prognostic value of left atrial volume index in degenerative mitral stenosis

**DOI:** 10.1007/s10554-022-02691-z

**Published:** 2022-07-18

**Authors:** Idit Yedidya, Steele C. Butcher, Jan Stassen, Pieter van der Bijl, Jinghao Nicholas Ngiam, Nicholas W. S. Chew, Ching-Hui Sia, Ryan Leow, Tony Yi-Wei Li, William K. F. Kong, Kian Keong Poh, Ran Kornowski, Nina Ajmone Marsan, Victoria Delgado, Jeroen J. Bax

**Affiliations:** 1 Department of Cardiology, Leiden University Medical Center, Albinusdreef 2, 2300 RC Leiden, The Netherlands; 2 Department of Cardiology, Rabin Medical Center, Petach-Tikva, Israel. Affiliated with the Faculty of Medicine, Tel-Aviv University, Tel Aviv, Israel; 3 Department of Cardiology, Royal Perth Hospital, Perth, Australia; 4 Department of Cardiology, Jessa Hospital, Hasselt, Belgium; 5 Department of Medicine, National University Health System Singapore, Singapore, Singapore; 6 Department of Cardiology, National University Heart Centre Singapore, Singapore, Singapore; 7 Turku PET Centre, University of Turku and Turku University Hospital, Turku, Finland

**Keywords:** Degenerative mitral stenosis, Prognosis, Left atrial volume index

## Abstract

**Purpose:**

Degenerative mitral stenosis (DMS) is associated with a poor prognosis. Although mean transmitral gradient (TMG) has shown a good correlation with outcome, little is known about the association between other echocardiographic parameters and prognosis in patients with DMS. The current study aimed to evaluate the prognostic value of left atrial volume index (LAVI) in patients with DMS.

**Methods:**

A total of 155 patients with DMS (72[63–80] years, 67% female) were included. The population was divided according to LAVI: normal-sized LAVI (LAVI ≤ 34 ml/m2); and enlarged LAVI (> 34 ml/m2).

**Results:**

Patients with enlarged LAVI had a higher left ventricular mass index (120[96–146] vs. 91[70–112] g/m2 p < 0.001), as well as a higher prevalence of significant mitral regurgitation and severe aortic stenosis (23% vs. 10% p = 0.046 and 38% vs. 15% p=0.001, respectively) compared to patients with normal-sized LAVI. During a median follow-up of 25 months, 56 (36%) patients died. Patients with enlarged LAVI had worse prognosis compared to patients with normal-sized LAVI (p = 0.026). In multivariable Cox regression model, an enlarged LAVI was independently associated with all-cause mortality (HR 2.009, 95% CI 1.040 to 3.880, P = 0.038).

**Conclusion:**

An enlarged LAVI (> 34 ml/m2) is significantly associated with excess mortality in patients with DMS. After adjusting for potential confounders, an enlarged LAVI was the only parameter that remained independently associated with prognosis.

**Supplementary Information:**

The online version contains supplementary material available at 10.1007/s10554-022-02691-z.

## Introduction

Mitral annular calcification (MAC) is a degenerative process arising from the mitral annulus and can extend to the mitral valve leaflets. This process may cause a reduction in effective mitral valve orifice area and failure of leaflet coaptation, resulting in degenerative mitral stenosis (DMS) [1]. The prognosis of DMS is poor, with a 5-year mortality rate of up to 50% [2]. However, not much is known about the echocardiographic prognostic parameters in patients with DMS. It has been shown that an increased mean transmitral gradient (TMG ≥ 2 mmHg) is associated with worse outcomes [2], with mortality increasing in parallel to the increase in mean TMG [3]. However, in patients with DMS, the mean TMG may not only be related to annular calcification and consequent narrowing of the mitral orifice area. An increased TMG may also be related to other conditions influencing the pressure gradient between the left ventricle and atrium (e.g. reduced left ventricular and/or atrial compliance), especially since patients with DMS are generally older and have multiple comorbidities including hypertension, left ventricular diastolic dysfunction, and atrial fibrillation [4], all of which may impact left atrial (LA) volume. Accordingly, the LA volume index (LAVI) may be an important factor related to prognosis in patients with DMS. Therefore, the aim of the current study is to evaluate whether an enlarged LAVI is associated with worse outcomes in patients with DMS.

## Methods

### Study population

Patients diagnosed with DMS, between February 2001 and October 2017 from the Leiden University Medical Center (Leiden, The Netherlands) and the National University Health System (Singapore) were selected. DMS was defined as MAC with a mean TMG ≥ 2 mmHg. Patients with previous mitral valve intervention or rheumatic mitral stenosis, were excluded. Demographic and clinical data (including AF, stroke, associated comorbidities, and medical therapy) were collected from hospital electronic patient files. For retrospective analysis of clinically acquired data, the institutional review board of each center waived the need for written patient informed consent. All data used for this study were acquired for clinical purposes and handled anonymously.

### Echocardiographic data acquisition and analysis

Transthoracic echocardiography images were recorded using commercially available ultrasound devices. Echocardiography was performed with patients at rest, in the left lateral decubitus position. Electrocardiogram-triggered echocardiographic data were acquired and digitally stored in cine-loop format for offline analysis.

Left ventricular end-diastolic and end-systolic volumes were measured in apical two- and four-chamber views, and the left ventricular ejection fraction (LVEF) was calculated using the biplane Simpson’s method. LA volume was measured on apical 2- and 4-chamber views using the biplane method and indexed to body surface area to calculate LAVI [4]. The population was divided into two groups based on LA size, according to recommendations for cardiac chamber quantification by echocardiography [4]: patients with normal-sized LAVI (≤ 34 ml/m^2^) and patients with enlarged LAVI (> 34 ml/m^2^).

Continuous spectral Doppler was used to measure the forward diastolic flow across the mitral valve (mean TMG) in the apical four-chamber view, and was averaged over 3 cardiac cycles in sinus rhythm and 5 cardiac cycles in AF [5]. The severity of mitral regurgitation was assessed following an integrative approach that includes qualitative, semiquantitative and quantitative measures according to contemporary guidelines.

The tricuspid annular plane systolic excursion was used for evaluation of right ventricular systolic function, measured on a right-ventricular focused apical 4-chamber view using M-mode. Concomitant aortic stenosis (AS) severity was determined by the evaluation of the peak aortic velocity, mean transaortic gradient, aortic valve area according to the continuity equation, and dimensionless valve index according to guideline recommendations [6, 7].

### Follow-up

The primary outcome of the study was all-cause mortality. Mortality data were collected via the departmental cardiology information system (EPD-Vision 11.8.4.0, Leiden University Medical Center, Leiden, The Netherlands), which is linked to the governmental registry of mortality, and the computerized patient support system (CPSS) in Singapore.

### Statistical analysis

Categorical data are presented as frequencies and percentages. Continuous data are expressed as mean ± standard deviation if normally distributed and median and interquartile range (IQR) if non-normally distributed. Categorical data were compared with the χ^2^ test. Normally distributed variables were compared using the independent samples t-test, while non-normally distributed variables were compared with the Mann–Whitney U test. Cumulative event-free survival for the endpoint of all-cause mortality was evaluated using the Kaplan–Meier method, and the log-rank test was used to compare groups. A multivariable Cox proportional hazards model was constructed to investigate the association of combined enlarged LAVI and mean TMG with all-cause mortality. Covariates considered to have a prognostic association on the basis of epidemiological data, including age, sex, mean TMG, LVEF ≥ 50% and hypertension, were included in the multivariable model. Hazard ratio (HR) and 95% confidence intervals (CI) were reported. Twente random individuals were selected for evaluation of interobserver agreement of LAVI, using intraclass correlation coefficients (ICCs) and Bland–Altman analysis. The second observer was blinded to the measurements of the first observer for interobserver measurements. All tests were two-sided and p-values < 0.05 were considered statistically significant. Statistical analysis was performed using SPSS version 25.0 (IBM Corporation, Armonk, New York) and R version 4.0.1 (R Foundation for Statistical Computing, Vienna, Austria).

## Results

### Patient population

A total of 155 patients (67% women, median age 72 years [IQR 63 to 80]) were included. Of these patients, 69% had hypertension, 32% had AF, and 24% had a history of stroke. A total of 67 patients had a normal-sized LAVI (≤ 34 ml/m^2^), whereas 88 patients had enlarged LAVI (> 34 ml/m^2^). Patients with enlarged LAVI had a higher prevalence of arterial hypertension and were using more medications than patients with normal-sized LAVI (Table 1).

**Table 1:** Baseline clinical characteristics

Variable	Overall population (n = 155)	LAVI ≤ 34 m/m^2^ (n = 67)	LAVI > 34 m/m^2^ (n = 88)	p-value
Age, years	72[63–80]	68[61–76]	78[67–83]	< 0.001
Female, n(%)	104(67)	44(66)	60(68)	0.742
BSA, m^2^	1.75[1.60–1.93]	1.70[1.55–1.89]	1.77[1.63–1.94]	0.195
Arterial hypertension, n(%)	103(69)	37(56)	66(80)	0.002
Diabetes mellitus, n(%)	44(30)	22(33)	22(27)	0.390
GFR < 30, ml/min/1.73 m^2^	17(12)	6(10)	11(14)	0.422
ß-blockers, n(%)	73(50)	24(37)	49(60)	0.006
Calcium channel blockers, n(%)	30(20)	12(19)	18(22)	0.602
ACEI/ARB, n(%)	61(42)	20(31)	41(50)	0.019
Diuretics, n(%)	57(40)	16(25)	41(49)	0.002
Anticoagulants, n(%)	37(25)	15(23)	22(27)	0.572
Atrial fibrillation, n(%)	48(32)	22(33)	26(31)	0.794
Stroke, n(%)	36(24)	15(23)	21(26)	0.685

Data are presented as mean ± SD if normally distributed or median (25th–75th percentile) if not normally distributed

*ACEI* angiotensin-converting enzyme inhibitor, *ARB* angiotensin receptor blocker, *BSA* body surface area, *GFR* glomerular filtration rate, *LAVI* left atrial volume index

### Echocardiographic characteristics

The overall study population had preserved left and right ventricular systolic function. A total of 84% had an LVEF ≥ 50% and the median tricuspid annular plane systolic excursion was 22 mm [IQR 19 to 23]. The median TMG was 6 mmHg [IQR 4 to 8], 60 (39%) patients had mean TMG < 5 mmHg, 75 (48%) mean TMG 5–10 mmHg and 20 (13%) mean TMG > 10 mmHg. Concomitant significant (≥ moderate) MR was found in 17% of the study population and 28% had severe AS. The median LAVI was 40 ml/m^2^ [IQR 28 to 52]. When dividing the population according to the presence of enlarged LAVI, the group of patients with enlarged LAVI had a higher left ventricular mass index as well as a higher prevalence of significant MR and severe AS compared to their counterparts (Table 2). The ICC for LAVI interobserver variability was 0.927(95% CI: 0.817–0.971), p < 0.001).

**Table 2:** Baseline echocardiographic characteristics

Variable	Overall population (n = 155)	LAVI ≤ 34 ml/m^2^ (n = 67)	LAVI > 34 ml/m^2^ (n = 88)	p-value
Heart rate, bpm	77[67–88]	77[67–90]	77[67–87]	0.976
LVEF ≥ 50%, n(%)	129(84)	59(88)	70(81)	0.261
LVMI, g/m^2^	105[82–132]	91[70–112]	120[96–146]	< 0.001
LVEDVI, ml/m^2^	49[39–61]	51[38–59]	48[40–65]	0.539
LVESVI, ml/m^2^	19[13–25]	19[12–25]	19[13–25]	0.449
LAVI, ml/m^2^	40[28–52]	27[25–31]	48[42–60]	< 0.001
TMG mean, mmHg	6[4–8]	5[4–9]	6[4–7]	0.973
Significant MR, n(%)	27(17)	7(10)	20(23)	0.046
Severe AS, n(%)	43(28)	10(15)	33(38)	0.001
AVA, cm^2^	1.44[0.95–2.49]	1.9[1.23–2.9]	1.2[0.8–1.83]	< 0.001
TAPSE, mm	22[19–23]	22[20–22]	21[18–25]	0.577
SPAP, mmHg	42 ± 12	41 ± 14	42 ± 11	0.613

Data are presented as mean ± SD if normally distributed or median (25^th^-75^th^ percentile) if not normally distributed

*AS* aortic stenosis, *AVA* aortic valve are; bpm; beats per minute, *LAVI* left atrial volume index, *LVEDVI* left ventricular end-diastolic volume index, *LVEF* left ventricular ejection fraction, *LVESVI* left ventricular end-systolic volume index, *LVMI* left ventricular mass index, *MR* mitral regurgitation, *SPAP* systolic pulmonary artery pressure, *TAPSE* tricuspid annular plane systolic excursion, *TMG* transmitral gradient

### Patients outcomes

**Fig. 1:**
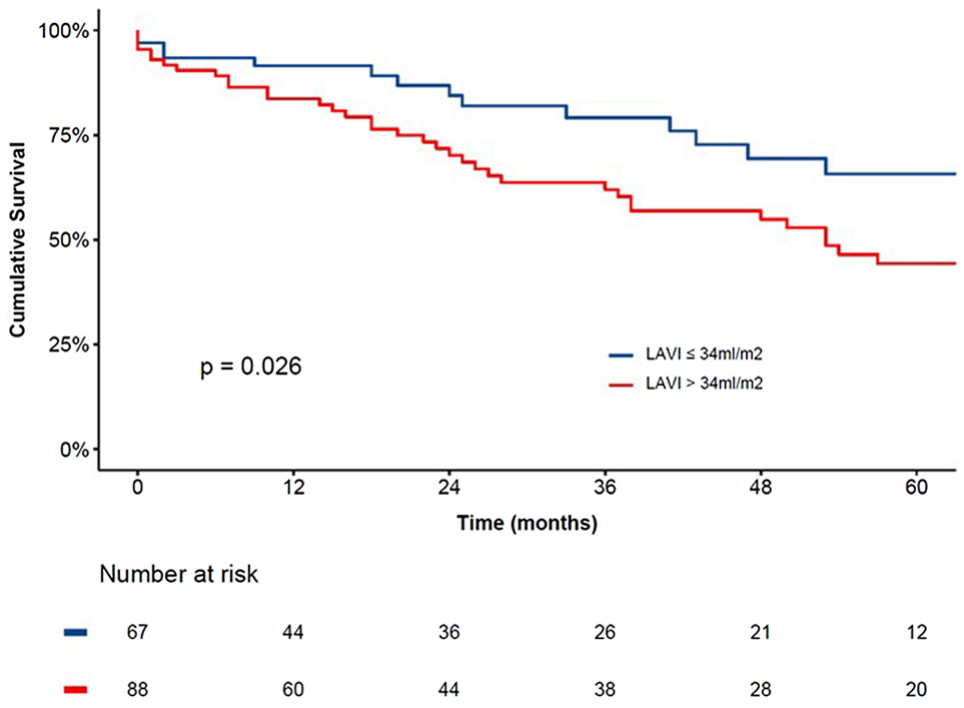
Kaplan–Meier survival curves for all-cause mortality. The Kaplan–Meier curves demonstrate the reduced survival of patients with enlarged LAVI (> 34 ml/m^2^) (red line) compared to patients with normal-sized LAVI (≤ 34 ml/m^2^) (blue line). *LAVI* left atrial volume index

During a median follow-up of 25 months [IQR 6–55], 56 (36%) patients died. The cumulative survival rates at 12, 24, and 60 months’ follow-up were superior for patients with normal-sized LAVI compared to patients with enlarged LAVI (92%, 84%, and 66%, vs. 84%, 70%, and 44%, respectively; p = 0.026; Fig. 1). Univariable Cox regression analysis demonstrated a significant association between enlarged LAVI and all-cause mortality (HR 1.937, 95% CI 1.069 to 3.509, p = 0.029). The ethnicity of the patient did not influence the outcome (p = 0.728). To evaluate the association between LAVI enlargement and outcomes in patients with DMS, a comprehensive multivariable Cox regression was performed. Three different models were constructed; model 1: adjusted enlarged LAVI with demographic variables including age and sex (HR 2.021, 95% CI 1.088 to 3.752, P = 0.026); model 2: included model 1 and echocardiographic variables as mean TMG and LVEF ≥ 50% (HR 2.105, 95% CI 1.128 to 3.930, p = 0.019); and model 3: included model 2 and the clinical variable of hypertension (HR 2.009 95% CI 1.040 to 3.880, p = 0.038). In all three models, enlarged LAVI remained independently associated with all-cause mortality (Table 3). A different model, included aortic valve area is available supplementary material (Table S2).

**Table 3:** Uni- and multivariable Cox regression analysis to investigate the association between LAVI enlargement and outcomes in patients with degenerative mitral stenosis

Variable	HR (95% CI)	p-value
LAVI (> 34 ml/m^2^)	1.937 (1.069–3.509)	0.029
Model 1: adjusted for age and sex	2.021 (1.088–3.752)	0.026
Model 2: Model 1 + mean TMG and LVEF ≥ 50%	2.105 (1.128–3.930)	0.019
Model 3: Model 2 + Hypertension	2.009 (1.040–3.880)	0.038

*LAVI* left atrial volume index, *LVEF* left ventricular ejection fraction, *TMG* transmitral gradient

## Discussion

The main findings of this study can be summarized as follows: (1) in patients with DMS, an enlarged LAVI is associated with older age and a higher prevalence of arterial hypertension, heart failure symptoms, heart failure medication and valvular heart disease (concomitant MR and severe AS); (2) patients with an enlarged LAVI have worse prognosis than patients with a normal-sized LAVI; and (3) an enlarged LAVI is independently associated with all-cause mortality after adjusting for demographic, clinical and echocardiographic risk factors.

### Clinical and echocardiographic variables associated with MAC formation

DMS is a consequence of MAC, a degenerative process of the mitral valve annulus that can extend to the mitral valve leaflets, thereby increasing the TMG. It has been considered mainly as a cardiac sign of aging [8, 9], with studies in these patients showing a median age > 70 years [2, 3]. Abnormal calcium-phosphorus metabolism, a common finding in patients with chronic renal disease, may contribute to MAC formation [10]. The process also has inflammatory pathophysiological mechanisms [11], Massera et al. showed that patients with MAC have evidence of increased local calcium activity and inflammation [12]. In a multivariable model, disease activity was associated with female sex. It remains unclear why female patients have a higher prevalence of MAC [13], with 67% female patients in the current study population. One hypothesis is the impact of medication to treat osteoporosis on the valve calcification process, which is already being investigated by the Scottish Aortic Stenosis and Lipid Lowering Trial, Impact on Regression (SALTIRE) II in patients with AS. Another theory has to do with sex hormones. Subramanya et al. found that an androgenic hormonal profile is associated with greater coronary artery calcium progression in post-menopausal women [14]. However, the impact of sex hormones on valvular calcification is poorly understood. Several hemodynamic conditions that cause an elevation in left ventricular peak end-systolic pressure can increase mitral annular tension and accelerate the degenerative process. Those conditions are mainly arterial hypertension, AS and left ventricular hypertrophy, and all of them were found to be associated with MAC [15, 16]. Those MAC risk factors have also been identified in our study population: two thirds of the current study population have arterial hypertension, one third severe AS, and the female patients had a high left ventricular mass index (106[78–128]g/m^2^).

### Echocardiographic variables associated with outcomes

For a long time, MAC has been considered as an innocent bystander of cardiac aging. In recent years, however, there has been growing evidence that patients with MAC and DMS have a worse prognosis. In 1004 patients with severe MAC and mean TMG ≥ 2 mmHg, Pasca et al. demonstrated that these patients had a very poor prognosis, with 1- and 5-year survival rates being 78% and 47%, respectively. Prognosis was worse in patients with a more advanced age, as well as in those with chronic kidney disease, AF and concomitant valvular lesions [2]. Another study by Bertrand et al., dividing 5754 patients with MAC into three groups according to the mean TMG, showed that survival rates decreased as the mean TMG increased [3]. Although prognostically relevant, mean TMG is not well validated in patients with DMS. Indeed, studies comparing echocardiographically-derived measurements of TMG with invasive hemodynamic measurements mainly included patients with rheumatic mitral valve stenosis [17]. Moreover, mean TMG is influenced by the pressure difference between the left ventricle and LA, and is therefore also influenced by reduced left ventricular compliance and an elevated LA pressure, conditions that often coexist in patients with DMS. In addition, mean TMG also depends on heart rate and stroke volume, which are affected by the high proportion of supraventricular arrhythmias seen in these patients. In contrast, LAVI is much less dependent on changes in cardiac hemodynamics or supraventricular arrhythmias and may better reflect the severity and duration of volume and pressure overload in patients with DMS or MR. A previous study already showed that patients with DMS had larger LAVI compared to a matched group of patients without DMS [5].

**Fig. 2:**
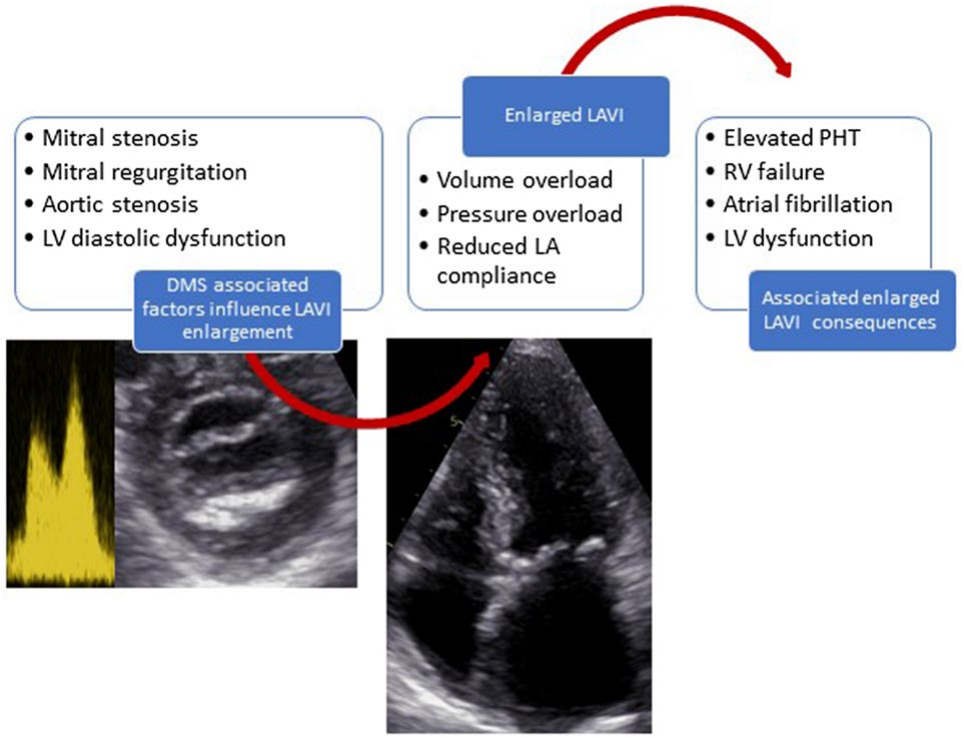
Algorithm of reduced prognosis associated with LAVI enlargement in patients with DMS. *DMS* degenerative mitral stenosis; *LA* left atrial, *LAVI* left atrial volume index, *LV* left ventricular, *PHT* pulmonary hypertension, *RV* right ventricular

In addition, LA dilatation has been shown to have a strong association with cardiovascular events and worse outcomes in different cardiovascular diseases [18, 19]. In 611 patients with rheumatic mitral stenosis, enlarged LAVI was independently associated with poor outcomes, although this association was less strong in patients with very severe mitral stenosis (mitral valve area ≤ 1 cm^2^) [20]. In the present study, LAVI enlargement (> 34 ml/m^2^) and aortic valve area were the only echocardiographic parameter that were associated with outcomes on univariable Cox regression analysis (Supplementary data – Table S1). LAVI remained independently associated with all-cause mortality after adjustment for other relevant confounders. Compared to the group with normal-sized LAVI, patients with enlarged LAVI had a significantly higher prevalence of comorbidities associated with MAC prognosis, including arterial hypertension, higher left ventricular mass index and severe AS (Fig. 2). This illustrates that comorbidities in patients with DMS may impact on LAVI. It remains difficult to distinguish between the impact of DMS and other cofactors on LA dilatation. The similar mean TMG in both groups supports the assumption that DMS probably contributes less to LAVI enlargement than the other comorbidities.

Assessment of the mitral valve area in patients with DMS is challenging. The different methods of measuring mitral valve stenosis are extrapolated from studies of rheumatic mitral stenosis and are less validated in patients with DMS. Moreover, there is a lack of information regarding the correlation of valve stenosis and mean TMG or prognosis in this patient population. Because LAVI is less influenced by instantaneous changes in heart rate or flow, and better integrates clinical and hemodynamic changes noted in patients with DMS, it probably better reflects the complexity of cardiac co-morbidities observed in these patients. Measurements of LAVI should therefore be included in the risk-stratification of patients with DMS.

### Study limitations

This is a retrospective, two centers study, with inherent limitations. Calcium burden and staging of the disease were not systematically available. Only one study center had strain analysis available, so this was not provided in the current study. The exact contribution of mitral valve area to LAVI enlargement could not be ascertained. Heart failure symptoms and coronary artery disease information were not included in the database. The study data was unsuitable for using the Charlson comorbidity index. Since the cause of death was not systematically ascertained, all-cause mortality was chosen as the endpoint. The study population was of limited size and larger studies are needed to confirm our observations.

## Conclusion

Greater LAVI is independently associated with all-cause mortality in patients with DMS. LAVI measurements may therefore be incorporated in the current risk stratification of these patients. Whether treatment of DMS will reduce LA dimensions and improve prognosis merits further investigation.

## Supplementary Information

Below is the link to the electronic supplementary material.

Supplementary file1 (DOCX 15 kb)

Supplementary file2 (DOCX 13 kb)
