# Supplementary material for: Prognostic value of left atrial volume index in degenerative mitral stenosis
Source: Int J Cardiovasc Imaging. 2022 Jul 18;38(12):2687–93. doi: 10.1007/s10554-022-02691-z (PMC9708792; doi:10.1007/s10554-022-02691-z)
Supplement: Supplementary file 1 — Supplementary file1 (DOCX 15 kb) [file 10554_2022_2691_MOESM1_ESM.docx]

**Supplementary data**

**Table S1. Univariable Cox regression analysis to investigate the association between LAVI enlargement and outcomes in patients with degenerative mitral stenosis**

| Variables | HR (95% CI) | P-value |
| --- | --- | --- |
| Demographic and clinical |  |  |
| Age | 1.002(0.980-1.025) | 0.855 |
| Female | 1.006(0.575-1.759) | 0.984 |
| Hypertension | 1.0507(0.785-2.894) | 0.217 |
| Diabetes mellitus | 0.951(0.518-1.747) | 0.872 |
| GFR<30 ml/m | 1.812(0.876-3.748) | 0.109 |
| Atrial fibrillation | 1.533(0.850-2.765) | 0.155 |
| NYHA Class II to IV | 1.700(0.943-3.064) | 0.077 |
| Echocardiographic |  |  |
| LVEF≥50% | 1.375(0.620-3.049) | 0.433 |
| LVMI | 0.995(0.987-1.003) | 0.198 |
| LVEDVI | 0.996(0.980-1.012) | 0.605 |
| LVESVI | 1.001(0.978-1.024) | 0.947 |
| LAVI >34 ml/m^2^ | 1.937(1.069-3.509) | 0.029 |
| LAVI | 1.014(0.998-1.029) | 0.079 |
| TMG mean | 1.046(0.958-1.142) | 0.320 |
| MR significant | 1.384(0.674-2.842) | 0.376 |
| Severe AS | 1.530(0.884-2.648) | 0.128 |
| AVA | 0.680(0.472-0.978) | 0.037 |
| TAPSE | 0.968(0.915-1.024) | 0.250 |

AS, aortic stenosis; AVA, aortic valve area; GFR, glomerular filtration rate; LAVI, left atrial volume index; LVEDVI, left ventricular end diastolic volume index; LVEF, left ventricular ejection fraction; LVESVI, left ventricular end systolic volume index; LVMI, left ventricular mass index; MR, mitral regurgitation; NYHA, New York Heart Association; TAPSE, tricuspid annular plane systolic excursion; TMG; transmitral gradient.
