# Supplementary material for: Prognostic value of left atrial volume index in degenerative mitral stenosis
Source: Int J Cardiovasc Imaging. 2022 Jul 18;38(12):2687–93. doi: 10.1007/s10554-022-02691-z (PMC9708792; doi:10.1007/s10554-022-02691-z)
Supplement: Supplementary file 2 — Supplementary file2 (DOCX 13 kb) [file 10554_2022_2691_MOESM2_ESM.docx]

| Multivariable Cox regression | HR (95% CI) | p-value |
| --- | --- | --- |
| LAVI>34 mm^2^  Age  Gender, female  TMG≥2mmHg  LVEF≥50%  AVA | 2.016(1.026-3.961)  0.992(0.968-1.017)  0.425(0.773-0.410)  1.113(0.707-1.754)  2.154(0.869-5.344)  0.680(0.465-0.995) | 0.042  0.529  0.425  0.643  0.098  0.047 |

**Table S2. Multivariable Cox regression analysis to investigate the association between LAVI enlargement and outcomes in patients with degenerative mitral stenosis**

AVA, aortic valve area; LAVI, left atrial volume index; LVEF, left ventricular ejection fraction; TMG, transmitral gradient.
